# “CRP-first” algorithm to guide imaging in suspected renal colic: a retrospective UK cohort study

**DOI:** 10.1007/s10140-025-02411-9

**Published:** 2025-11-08

**Authors:** Sayed Borna Farzaneh, Edward Antram, Sarah Naunton, Arpan Patel, Obi Ajuluchukwu, Naren Govindarajah, Lakshmi Ratnam, Marco Bolgeri, Nirav Patel, Anita Wale

**Affiliations:** 1 Department of Radiology, St George’s University Hospitals NHS Foundation Trust, London, UK; 2 Department of Urology, St George’s University Hospitals NHS Foundation Trust, London, UK; 3 City St George’s, University of London, London, UK

**Keywords:** Renal colic, C-reactive protein, Diagnostic accuracy, CT KUB, CT abdomen and pelvis, Triage

## Abstract

**Purpose:**

To evaluate whether admission C-reactive protein (CRP) can triage patients with suspected renal colic to low dose non contrast CT KUB or contrast enhanced CT of the abdomen and pelvis (CTAP) at first presentation.

**Methods:**

Retrospective single centre diagnostic accuracy study in a United Kingdom emergency department. Index test was admission CRP with a prespecified cut-off of 5 mg/L (positive if CRP ≥ 5 mg/L). Reference standard was CT classified a priori as: A normal, B simple calculus, C complicated calculus, D alternative acute diagnosis. The target condition for accuracy analyses was C or D. We constructed a 2 × 2 table and calculated sensitivity, specificity, predictive values and likelihood ratios with 95% confidence intervals.

**Results:**

Of 1,096 CT examinations during the study window, 233 were for suspected renal colic; 58 patients met eligibility and had admission CRP available (29 with CRP < 5 mg/L and 29 with CRP ≥ 5 mg/L). The target condition was present in 26/58 (44.8%). Using CRP ≥ 5 mg/L, sensitivity was 0.73 (95% CI 0.54–0.86), specificity 0.69 (0.51–0.82), positive predictive value 0.66 (0.47–0.80), negative predictive value 0.76 (0.58–0.88), likelihood ratio positive 2.34 (1.16–4.70) and likelihood ratio negative 0.39 (0.20–0.77).

**Conclusion:**

CRP provided modest but clinically interpretable probability shifts for complicated stones or alternative acute pathology. A CRP first approach may support initial imaging selection between CTAP and CT KUB. Prospective multicentre validation is required.

**Supplementary Information:**

The online version contains supplementary material available at 10.1007/s10140-025-02411-9.

## Key messages

What is already known on this topic – Low dose CT KUB is the recommended first line test for suspected renal colic, yet 10–71% of studies reveal alternative diagnoses, some of which require contrast CTAP and different management.

What this study adds – A CRP threshold of 5 mg/L distinguishes patients who are more likely to have complicated stones or alternative acute pathology.

How this study might affect research, practice or policy – Embedding a CRP first rule could reduce contrast exposure, radiation dose and diagnostic delay.

**Supplementary Information:**

The online version contains supplementary material available at 10.1007/s10140-025-02411-9.

## Introduction

Acute renal colic is a familiar presentation in the emergency department, affecting approximately 1–2 individuals per 1,000 each year [1]. The underlying cause is usually urinary tract obstruction by calculi, a condition that will be experienced at least once in the lifetime of about 12% of men and 6% of women [2]. Patients characteristically report severe, intermittent flank pain radiating towards the groin or lower abdomen and may also experience nausea, vomiting or visible or microscopic haematuria.

Non contrast computed tomography of the kidneys, ureters and bladder (CT KUB) is considered the first line investigation by the Royal College of Radiologists iRefer guidelines [3], offering sensitivities of 94–98% and specificities of 95–100% for stone detection [4]. In North America, the American College of Radiology (ACR) Appropriateness Criteria likewise support non-contrast CT as the most appropriate initial test for suspected urolithiasis, reserving contrast-enhanced CT when alternative diagnoses or complications are suspected [5]. 

Nevertheless, 10–71% of CT KUB examinations reveal alternative diagnoses, and roughly 12% are clinically significant [6–9]. Streamlining the initial triage of flank pain could therefore reduce radiation dose, shorten diagnostic delay and improve downstream management.

Inflammatory biomarkers have been explored as adjuncts to imaging selection. Prior studies report that white cell count, and neutrophil count are higher in acute appendicitis than in renal colic [10], and raised inflammatory markers form components of risk scores such as the Adult Appendicitis Score [11]. To date, no study has assessed whether an acute phase reactant can help discriminate uncomplicated renal colic from conditions that warrant contrast CT at first presentation. C reactive protein (CRP) is produced by the liver in response to inflammation, infection and tissue injury and is routinely available in emergency departments [12].

We aimed to determine whether serum CRP can predict the presence of complicated calculus or alternative pathology in patients with suspected renal colic. Our goal was to guide the choice of CT imaging. We hypothesised that a CRP threshold of 5 mg/L would identify patients suitable for initial imaging with CT KUB, while directing those at higher risk to contrast enhanced CTAP.

## Methods

### Study design and setting

We carried out a retrospective, single centre diagnostic accuracy study in the Emergency Department of St George's University Hospitals NHS Foundation Trust, London, United Kingdom. The research protocol was approved by the City St George's, University of London Ethics Committee (reference AU0049, 29 August 2023) with a waiver of informed consent granted due to its retrospective design. Patients or members of the public were not involved in the design, conduct, reporting or dissemination plans of this research.

### Patient identification

The Radiology Information System was interrogated for all low dose CT KUB and contrast enhanced CT abdomen and pelvis examinations performed on the emergency department scanner between 1 November 2022 and 1 March 2023. One investigator, blinded to outcome, screened request forms and electronic notes to include only cases in which renal colic was suspected, defined by explicit wording or compatible symptoms such as flank pain or hematuria. At our center CRP is typically ordered in the presence of fever/rigors, pain, peritonism or diagnostic uncertainty.

### Exclusion criteria

Non acute indications, absence of admission CRP, known stable renal stones, or contraindication to CT including pregnancy. Pregnancy was a prespecified exclusion because CT is generally avoided in pregnancy. During the study window, no pregnant patient otherwise meeting inclusion criteria underwent CT. We acknowledge that physiological changes in pregnancy may influence CRP values.

## Index test and reference standard

Assignment of CT categories (A–D) for the reference standard was performed by a radiologist who was blinded to admission CRP and other biochemical values; routine clinical information on the request was available as per standard reporting practice.

Index test: admission CRP measured in the emergency department. A prespecified threshold of 5 mg/L defined a positive result (CRP ≥ 5 mg/L) according to local laboratory upper reference limit for normal. The 5 mg/L cut-off was also prespecified based on a preliminary audit prioritizing sensitivity for urgent pathology. This decision is supported by Coyle et al. [13] who evaluated CRP across thresholds including 5 mg/L, supporting the pragmatism of using the laboratory “normal” boundary as an initial decision point.

Reference standard: CT was classified into four mutually exclusive categories:

- A normal.
- B simple calculus (renal or no ureteric stone without evidence of obstruction and no features of infection).
- C complicated calculus (ureteric or renal stone with obstructive uropathy and/or evidence of infection such as pyelonephritis, or perinephric inflammatory change).
- D alternative acute diagnosis (e.g. diverticulitis, appendicitis, tumor).

For diagnostic accuracy analyses, the target condition was category C or D. Non-obstructing renal or ureteric stones are included within simple calculus (Category B). Such cases can be symptomatic and because CRP often remains normal in the absence of obstruction or infection, a CRP-first step is less informative for these presentations [14]. 

### Imaging technique

All scans were acquired on a 128 slice Siemens SOMATOM Drive scanner adjacent to the emergency department. Low dose CT KUB used helical acquisition from the superior renal poles to the urinary bladder (typical dose length product 177 mGy·cm). Single phase CT abdomen and pelvis used weight based Omnipaque 300 with coverage from diaphragm to pubic symphysis (dose length product ~ 402 mGy·cm).

## Biochemical data

The index test (admission CRP) and the reference standard CT were obtained during the same emergency department attendance; the exact time interval between blood sampling and CT acquisition was not recorded.

Admission CRP and white cell count were retrieved from the electronic patient record. CRP was measured on a Roche Cobas c303 analyser; white cell count on a Beckman Coulter DxH 900 analyzer. The upper reference limit for normal for this assay is < 5 mg/L.

## Sample size and statistical analysis

Cases without admission CRP were excluded per eligibility criteria; all CT examinations could be classified into categories A–D and no indeterminate reference standard results occurred.

A preliminary audit of 82 attendances suggested that a CRP threshold of 5 mg/L optimised sensitivity for detecting alternative pathology. We inspected CRP as a continuous variable (Fig. 2) but prespecified 5 mg/L as the primary threshold. Cases were ordered chronologically and the first 29 with CRP < 5 mg/L and the first 29 with CRP ≥ 5 mg/L were selected. We constructed a 2 × 2 table and calculated sensitivity, specificity, positive and negative predictive values and likelihood ratios with 95% confidence intervals. Categorical variables were compared with Fisher’s exact test. CRP values were compared with the Mann–Whitney U test. Odds ratios with 95% confidence intervals were calculated for secondary patient outcomes. Analyses were performed using IBM SPSS Statistics v29. The STARD checklist will be submitted as a supplementary file.

## Addressing potential bias and applicability

We report recruitment and testing pathways descriptively here. Interpretive considerations regarding selection and applicability are presented in the Discussion.

## Results

**Fig. 1:**
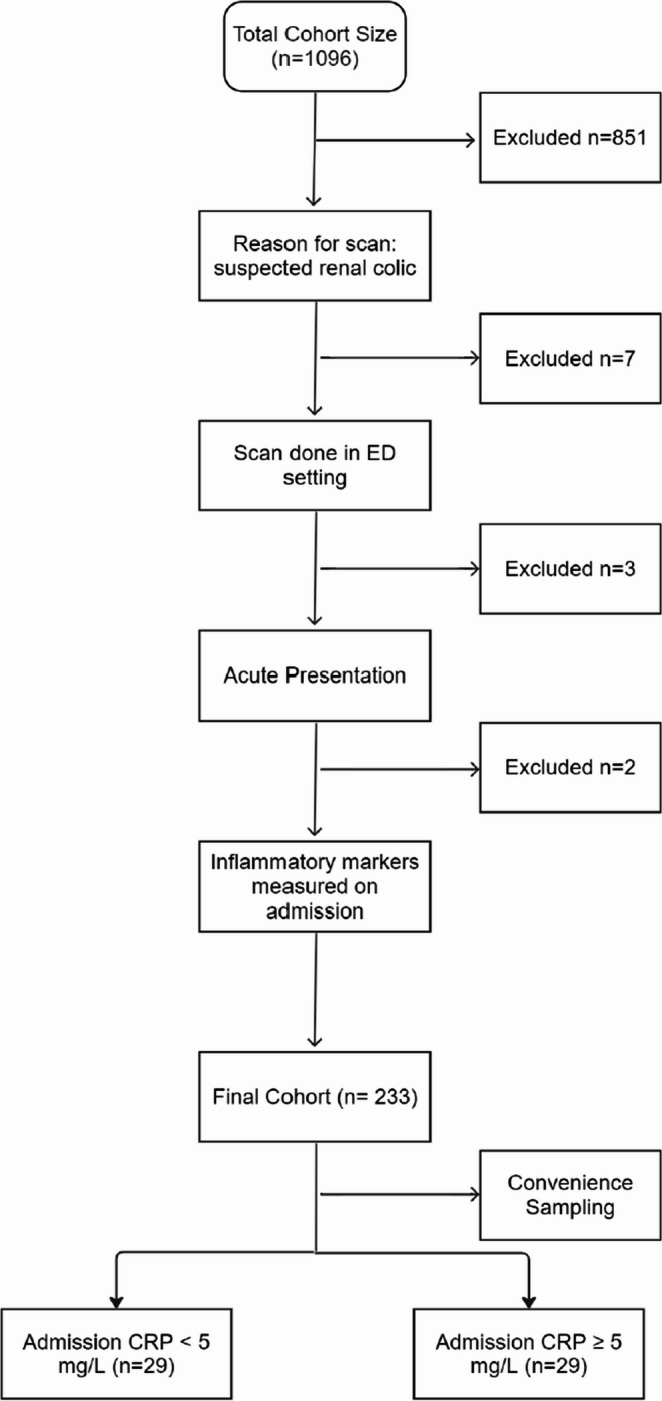
Participant flow diagram

**Fig. 2:**
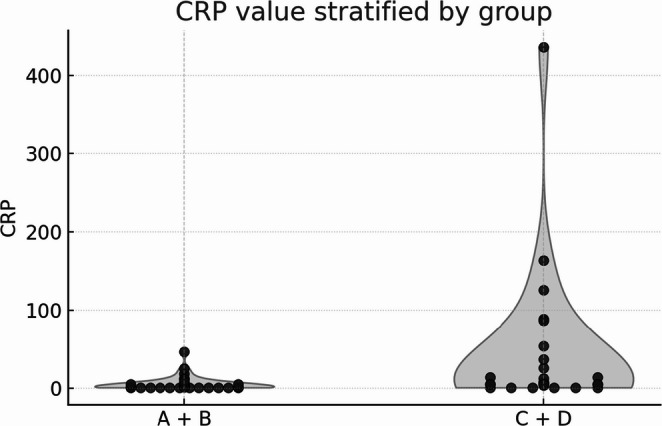
Violin plot of CRP values stratified by imaging category groups

A total of 1,096 patients underwent CT between 01/11/2022 and 01/03/2023. Clinical indication screening identified 233 scans for suspected renal colic. Applying the inclusion and exclusion criteria yielded 58 eligible patients. Participant flow is shown in Fig. 1. Baseline characteristics are summarized in Table 1, and the distribution of complicated calculi and alternative diagnoses is detailed in Table 2. The distribution of CRP values across imaging categories is shown in Fig. 2. The cross-tabulation of CRP and the target condition is presented in Table 3, with diagnostic accuracy estimates summarized in Table 4.

**Table 1:** Baseline patient characteristics and CT categories by CRP group

Variable	CRP < 5 mg L⁻¹ (*n* = 29)	CRP ≥ 5 mg L⁻¹ (*n* = 29)	*p*-value	Total (%)
Age, years – mean ± SD (range)	45 ± 11 (26–71)	46 ± 14 (24–83)	0.78	
Male sex, n (%)	17 (59%)	14 (48%)	0.43	
Group A – Normal study	17 (58.6%)	9 (31.0%)	0.064	26 (44.8%)
Group B – Simple calculus	5 (17.2%)	1 (3.4%)	0.19	6 (10.3%)
Group C – Complicated calculus	4 (13.8%)	7 (24.1%)	0.5	11 (19.0%)
Group D – Alternative diagnosis	3 (10.3%)	12 (41.4%)	**0.015**	15 (25.9%)
Groups A & B -	22	10	**0.003**	32
Groups C & D	7	19	**0.003**	26

**Table 2:** Breakdown of complicated calculi or alternative diagnosis (*n* = 26)

Group	Category	Specific diagnoses	*n*
**C**	**Complicated calculus**	Obstructing stone with hydronephrosis (7); calculus + pyelonephritis (3); calculus + perinephric stranding (1)	11
**D**	**Gastro-intestinal**	Acute diverticulitis (3); appendicitis (1); intra-abdominal collection (1); acute cholecystitis (1)	6
**D**	**Renal** **(non-urolithic)**	Hydroureter/hydronephrosis without stone (2); acute pyelonephritis without stone (2)	4
**D**	**Tumor**	Renal tumor (2); adrenal tumor (1)	3
**D**	**Gynecological**	Uterine fibroid	1
**D**	**Other**	Mesenteric lymphadenopathy; sclerotic bone lesions	1

**Table 3:** Diagnostic contingency table for CRP at 5 mg/L against target condition (categories C or D)

	Target condition absent (A or B) – normal/uncomplicated calculus	Target condition present (C or D) – severe acute finding
CRP < 5 mg/L (negative)	22	7
CRP ≥ 5 mg/L (positive)	10	19

**Table 4:** Diagnostic accuracy for CRP ≥ 5 mg/L detecting categories C or D

Measure	Estimate	95% CI
Sensitivity	73.1%	54.0–86.3%
Specificity	68.8%	51.4–82.0%
Positive predictive value	65.5%	47.3% to 80.1%
Negative predictive value	75.9%	58.9% to 87.8%
Likelihood ratio positive	2.34	1.16 to 4.70
Likelihood ratio negative	0.39	0.20 to 0.77

There was no significant difference in the number of patients with normal versus elevated CRP in Groups A to C, but there was in Group D with a higher proportion of elevated CRP (*p* = 0.015). Altogether, 26 of 58 patients (44.8%) fell into Groups C and D. Nineteen of 26 (73.1%) had a high CRP compared to 7 of 26 (26.9%) with a normal CRP (*p* = 0.002). Using 5 mg/L as the threshold for a high CRP, sensitivity for severe acute finding was 73% (95% CI 54–86%), specificity 69% (51–82%), positive predictive value 66% (47–81%) and negative predictive value 76% (58–88%). Likelihood ratio positive was 2.34 (95% CI 1.16–4.70) and likelihood ratio negative was 0.39 (0.20–0.77).

All patients with Gastrointestinal pathology had CRP ≥ 5 mg/L. 7 patients had CRP < 5 mg/L yet CT evidence of complicated or alternative pathology (false negatives), 3 of these had mild obstruction, 1 had early inflammatory change without systemic response and the other 3 showed non-specific findings requiring further imaging. None required emergency intervention or were admitted.

### Patient outcomes

Elevated CRP conferred a 3.1 fold increased risk of any acute finding (Groups B, C or D) and a 6.0 fold increased risk of a severe acute finding (Groups C or D). CRP elevation was not associated with a higher probability of repeat imaging within 7 days (OR 2.1, *p* = 0.56). White cell count showed no discriminative value (*p* = 0.38).

## Discussion

Our findings show that even a modest rise in CRP (≥ 5 mg L⁻¹) has clear implications across the diagnostic pathway. Complicated stones or alternative non urolithic conditions (Groups C and D) accounted for 44.8% of all presentations, and 65.5% with a raised CRP fell into these higher risk categories, compared with about one quarter of those whose CRP was normal (*p* = 0.002).

At a threshold of 5 mg L⁻¹, CRP had a sensitivity of 73% and a negative predictive value of 76% for detecting any complicated calculus or alternative diagnosis. These correspond to likelihood ratio positive 2.34 and likelihood ratio negative 0.39, which are clinically interpretable for first-line imaging selection. Thus, patients with a low CRP are unlikely to harbour an urgent finding and can, in most cases, proceed directly to low dose CT KUB without the need for further imaging.

A clear gradient of risk was observed: a raised CRP tripled the odds (OR 3.1, *p* = 0.04) of any acute finding (Groups B, C or D) and increased the odds (OR 6.0, *p* = 0.002) of a severe acute finding (Groups C or D) six fold. Despite this, CRP did not predict the need for repeat imaging within seven days, suggesting its utility lies chiefly in the initial imaging decision rather than in follow-up planning. This may also reflect radiologists’ reluctance to repeat imaging unless necessary. White cell count offered no such discrimination (*p* = 0.38), underscoring CRP as the more informative inflammatory marker for triage. Alternative diagnoses were more frequent in the elevated-CRP cohort (Table 1). Within Group D, the pattern was predominantly gastrointestinal (diverticulitis/appendicitis) with smaller numbers of renal non-urolithic, tumor and other entities (Table 2); this is consistent with CRP tracking inflammatory pathology, while neoplastic/other categories show greater heterogeneity in CRP.

No previous study has evaluated CRP expressly to guide the choice between low dose CT KUB and contrast enhanced CT of the abdomen and pelvis (CTAP). Angulo et al. [15] employed a higher cut off (28 mg L⁻¹) to predict the need for ureteric stenting, reporting 76% sensitivity and 89% specificity [15]. Our lower threshold, matching clinical practice, is geared towards sensitivity so that clinically significant disease is not missed; future work could explore tiered values, for example < 5, 5–20 and >20 mg L⁻¹, to refine specificity without compromising safety.

Our findings are consistent with ED literature showing that low CRP (0–5 mg/L) does not exclude positive abdomino-pelvic CT findings and that higher CRP increases yield (Coyle et al. [13]). Accordingly, prospective renal-colic studies should model CRP continuously and evaluate clinically anchored tiers (e.g., < 5, 5–20, >20 mg/L) within pre-specified multivariable frameworks, rather than re-deriving a single optimal cut-off in a small dataset. All cases of acute gastro-intestinal pathology in our series occurred in the CRP high cohort, mirroring surgical data that link elevated CRP with appendicitis and diverticulitis [10]. A “CRP first” algorithm could therefore stream low CRP patients straight to low dose CT KUB whilst directing those with a raised CRP to single phase CTAP. Such an approach shortens diagnostic delay, reduces cumulative radiation exposure (≈ 4.7 mSv versus ≈ 16 mSv) and limits contrast administration to the patients most likely to benefit [16–18]. The small number of clinically mild false negatives suggests that a CRP-first approach is safe only when integrated with clinical review: imaging should still proceed if pain persists, infection is suspected or examination findings are concerning.

Strengths of our study include a prespecified threshold informed by preliminary audit data, blinded CT classification using clearly defined categories, and standardised imaging protocols, all of which support internal validity.

Several limitations should be noted. CRP testing was performed at clinician discretion rather than universally so the analyzed cohort may over-represent diagnostically complex or higher-risk presentations. This case mix could inflate apparent specificity and PPV relative to an unselected population. CRP is a non-specific marker that rises with chronic inflammatory conditions and advancing age [19, 20]. Because we did not capture exact time interval between CRP sampling and CT, evolving inflammation could lead to misclassification. This uncertainty likely attenuates associations between CRP and imaging outcomes.

We did not fit data-driven thresholds or multivariate models owing to overfitting risk in this small retrospective cohort. Prospective validation should model CRP continuously within pre-specified multivariate frameworks (with adequate events-per-variable) to obtain transportable estimates. Generalizability is therefore to similar ED workflows and CRP practices. A prospective pathway with universal CRP sampling or random sampling, pre-specified imaging triggers and blinded interpretation would be needed to obtain unbiased accuracy estimates. Cost-effectiveness and radiation-dose modelling were beyond the scope of the present work. However, we have shown that utilizing the laboratory assay thresholds to determine choice of imaging is effective.

Diagnostic performance cannot be extrapolated to pregnancy. Physiological variation in CRP during pregnancy may reduce specificity at a 5 mg/L threshold, so any CRP-first approach would require pregnancy-specific validation. Whilst pregnancy is not an absolute contraindication to CT imaging therefore non-ionizing imaging techniques should be employed thus making CRP thresholds less important in this cohort. CRP is non-specific and may be elevated at baseline in chronic inflammatory conditions, malignancy, chronic infection and with increasing age. We did not adjust for comorbidities or age, which may reduce specificity of a 5 mg/L threshold.

A multicenter prospective study using the described methodology could validate the 5 mg L⁻¹ cut off and investigate stratified thresholds. Integrating clinical features such as fever, urinalysis or point-of-care ultrasound, and where appropriate, existing stone-risk scores adds incremental value over CRP alone. Future research should also address economic outcomes, including length of stay and time to definitive therapy, to quantify clinical and economic value.

In summary, a “CRP-first” imaging algorithm aligns diagnostic resources with clinical risk. Even a modest elevation (≥ 5 mg L⁻¹) increases the likelihood of pathology caused by complicated renal stones or conditions that are non-urological in origin, while a low CRP identifies patients who can begin, and often end, the diagnostic pathway with low dose CT KUB.

## Supplementary Information

Below is the link to the electronic supplementary material.

Supplementary Material 1(DOCX 29.6 KB)

## Data Availability

Available from the corresponding author on reasonable request.

## Declarations

### Patient and public involvement

Patients or members of the public were not involved in the design, conduct, reporting or dissemination plans of this research.

### Protocol availability

The analysis plan and protocol are available from the corresponding author on reasonable request.
