# Supplementary material for: “CRP-first” algorithm to guide imaging in suspected renal colic: a retrospective UK cohort study
Source: Emerg Radiol. 2025 Nov 8;32(6):939–45. doi: 10.1007/s10140-025-02411-9 (PMC12700957; doi:10.1007/s10140-025-02411-9)
Supplement: Supplementary file 1 — Supplementary Material 1(DOCX 29.6 KB) [file 10140_2025_2411_MOESM1_ESM.docx]

# STARD 2015 Checklist – Completed for “CRP-first algorithm to guide imaging in suspected renal colic”

| Section & Topic | No | Item | Reported (page/section/figure/table) |
| --- | --- | --- | --- |
| TITLE/ABSTRACT | 1 | Identify as a diagnostic accuracy study with at least one accuracy measure. | Abstract – Methods/Results (sensitivity, specificity, LRs reported). |
| ABSTRACT | 2 | Structured summary of study design, methods, results, conclusions. | Structured Abstract (Purpose, Methods, Results, Conclusion). |
| INTRODUCTION | 3 | Scientific and clinical background; intended use and clinical role of index test. | Introduction – rationale and triage role for CRP. |
| INTRODUCTION | 4 | Study objectives and hypotheses. | Introduction – final paragraph. |
| METHODS – Study design | 5 | Prospective or retrospective. | Methods – Study design and setting (retrospective). |
| METHODS – Participants | 6 | Eligibility criteria. | Methods – Exclusion criteria; Patient identification. |
| METHODS – Participants | 7 | Basis for identifying potentially eligible participants. | Methods – Patient identification (ED CTs; symptoms/requests). |
| METHODS – Participants | 8 | Where and when participants were identified. | Methods – Study design and setting (St George’s ED; 1 Nov 2022–1 Mar 2023). |
| METHODS – Participants | 9 | Consecutive, random or convenience series. | Methods – Sample selection (chronological selection of first 29 per CRP stratum; convenience). |
| METHODS – Test methods | 10 | Index test details to allow replication. | Methods – Index test; Biochemical data (Roche Cobas c303). |
| METHODS – Test methods | 10b | Reference standard details to allow replication. | Methods – Reference standard; Imaging technique (Siemens SOMATOM Drive; protocols). |
| METHODS – Test methods | 11 | Rationale for choosing the reference standard. | Methods – Reference standard (CT as best available; national guidance). |
| METHODS – Test methods | 12a | Definition/rationale for index test cut-offs (pre-specified vs exploratory). | Methods – Index test; Sample size/statistical analysis (pre-specified CRP ≥ 5 mg/L from audit). |
| METHODS – Test methods | 12b | Definition/rationale for reference standard result categories. | Methods – Reference standard (A–D; target condition C or D). |
| METHODS – Test methods | 13a | Availability of clinical info/reference standard to index test performers/readers. | Not applicable to lab processing; routine lab blinded to CT results (implicit). |
| METHODS – Test methods | 13b | Availability of clinical info/index test results to assessors of the reference standard. | Methods – Added statement: radiologist blinded to CRP; routine request info available. |
| METHODS – Analysis | 14 | Methods for estimating/comparing accuracy. | Methods – Statistical analysis (2×2 table; CIs; tests). |
| METHODS – Analysis | 15 | Handling indeterminate index or reference standard results. | Methods – Added statement: no indeterminate CT categories; CRP indeterminate not applicable. |
| METHODS – Analysis | 16 | Handling missing data on index test/reference standard. | Methods – Exclusion of cases without CRP (eligibility). |
| METHODS – Analysis | 17 | Analyses of variability in accuracy (pre-specified vs exploratory). | Not performed (stated in limitations). |
| METHODS – Analysis | 18 | Intended sample size and how determined. | Methods – Sample size/statistical analysis (stratified first 29 per CRP stratum from audit). |
| RESULTS – Participants | 19 | Flow of participants, using a diagram. | Results – Participant flow; Figure 1 (CONSORT-style flow). |
| RESULTS – Participants | 20 | Baseline demographic/clinical characteristics. | Results – Table 1. |
| RESULTS – Participants | 21a | Distribution of disease severity in those with target condition. | Results – Table 2 (complicated calculi subtypes). |
| RESULTS – Participants | 21b | Distribution of alternative diagnoses in those without the target condition. | Results – Table 1 (A/B categories). |
| RESULTS – Participants | 22 | Time interval and any clinical interventions between index and reference standard. | Methods – Added statement: same ED attendance; interval not recorded. |
| RESULTS – Test results | 23 | Cross-tabulation of index test by reference standard. | Results – Table 3. |
| RESULTS – Test results | 24 | Estimates of accuracy and precision (e.g., 95% CIs). | Results – Table 4. |
| RESULTS – Test results | 25 | Adverse events from performing the index test or reference standard. | Results – Added statement: none identified. |
| DISCUSSION | 26 | Study limitations, sources of bias, uncertainty, generalisability. | Discussion – Limitations paragraph. |
| DISCUSSION | 27 | Implications for practice; intended use and clinical role. | Discussion – Implications for triage and imaging selection. |
| OTHER INFORMATION | 28 | Registration number and name of registry. | Not registered (retrospective diagnostic accuracy study). |
| OTHER INFORMATION | 29 | Where the full study protocol can be accessed. | Statements – Added protocol availability on request. |
| OTHER INFORMATION | 30 | Sources of funding and other support; role of funders. | Statements – Funding: none; no role. |
